# NQO1 as a predictor of response to adjuvant GemCap treatment for pancreatic cancer

**DOI:** 10.1093/jnci/djaf345

**Published:** 2025-12-01

**Authors:** Dylan Williams, Chandni Patel, Kate Murray, Lucy Oldfield, Benjamin Small, Lawrence N Barrera, Rachel O’Sullivan, James Birch-Ford, Anthony Evans, Fiona Campbell, Pedro A Perez-Mancera, Christopher M Halloran, Daniel Palmer, William Greenhalf, Ian M Copple, Chris Goldring, Thilo Hackert, Richard Jackson, John P Neoptolemos, Christoph Springfeld, Markus W Büchler, Christoph W Michalski, Paula Ghaneh, Eithne Costello

**Affiliations:** Liverpool Experimental Cancer Medicine Centre, University of Liverpool, Liverpool, United Kingdom; Faculty of Health Social Care & Medicine, Edge Hill University, Ormskirk, United Kingdom; Liverpool Experimental Cancer Medicine Centre, University of Liverpool, Liverpool, United Kingdom; Liverpool Experimental Cancer Medicine Centre, University of Liverpool, Liverpool, United Kingdom; Liverpool Experimental Cancer Medicine Centre, University of Liverpool, Liverpool, United Kingdom; Liverpool Experimental Cancer Medicine Centre, University of Liverpool, Liverpool, United Kingdom; Liverpool Experimental Cancer Medicine Centre, University of Liverpool, Liverpool, United Kingdom; Liverpool Experimental Cancer Medicine Centre, University of Liverpool, Liverpool, United Kingdom; Liverpool Experimental Cancer Medicine Centre, University of Liverpool, Liverpool, United Kingdom; Liverpool Experimental Cancer Medicine Centre, University of Liverpool, Liverpool, United Kingdom; Liverpool Experimental Cancer Medicine Centre, University of Liverpool, Liverpool, United Kingdom; Liverpool Experimental Cancer Medicine Centre, University of Liverpool, Liverpool, United Kingdom; Liverpool Experimental Cancer Medicine Centre, University of Liverpool, Liverpool, United Kingdom; Liverpool Experimental Cancer Medicine Centre, University of Liverpool, Liverpool, United Kingdom; Liverpool Experimental Cancer Medicine Centre, University of Liverpool, Liverpool, United Kingdom; Department of Pharmacology and Therapeutics, University of Liverpool, Liverpool, United Kingdom; Department of Pharmacology and Therapeutics, University of Liverpool, Liverpool, United Kingdom; Department of General, Visceral and Thoracic Surgery, University Hospital Hamburg-Eppendorf, Hamburg, Germany; Liverpool Experimental Cancer Medicine Centre, University of Liverpool, Liverpool, United Kingdom; Department of General Surgery, University of Heidelberg, Heidelberg, Germany; Department of Medical Oncology, National Center for Tumor Diseases, Heidelberg University Hospital, Heidelberg, Germany; Department of General Surgery, University of Heidelberg, Heidelberg, Germany; Department of General, Visceral and Thoracic Surgery, University Hospital Hamburg-Eppendorf, Hamburg, Germany; Liverpool Experimental Cancer Medicine Centre, University of Liverpool, Liverpool, United Kingdom; Liverpool Experimental Cancer Medicine Centre, University of Liverpool, Liverpool, United Kingdom

## Abstract

**Background:**

NAD(P)H quinone dehydrogenase 1 (NQO1), a detoxification enzyme regulated by the Nrf2 cytoprotective pathway, is overexpressed in pancreatic ductal adenocarcinoma (PDAC). NQO1 levels are also influenced by the C609T single-nucleotide polymorphism (SNP). We hypothesized that elevated NQO1 would confer chemoresistance in PDAC and predict poor patient outcome.

**Methods:**

NQO1 tumor levels and germline C609T SNP status were assessed in archival samples from the European Study Group for Pancreatic Cancer (ESPAC) trials. NQO1 expression (H-score) was treated as continuous for survival regression analyses and dichotomized for visual summaries. Nrf2 or downstream gene induction was assessed in Nrf2 reporter mice or in PDAC cells following exposure to gemcitabine (Gem), 5-fluorouracil (5-FU), or the capecitabine (Cap) metabolite 5-fluoro-5′-deoxyuridine (5’-DFUR). Colony formation following NQO1 depletion was assessed.

**Results:**

NQO1 tumor levels correlated with germline C609T SNP status (*P* < .001). Contrary to our hypothesis, high NQO1 expression was associated with improved survival in ESPAC-4 patients randomized to GemCap (HR = 0.87 [95% CI = 0.751 to 0.999]; *P* = .049), and had no association to outcome in the Gem-only treated arm (HR = 0.98 [95% CI = 0.78 to 1.23]; *P* = .867). Including genotype data did not improve predictive model performance. Neither Gem nor 5-FU induced Nrf2 in vivo. At high concentrations, they suppressed Nrf2/NQO1 in PDAC cells, an effect not mitigated by co-treatment with 5’-DFUR. NQO1 depletion experiments revealed that NQO1 inhibits colony formation. The strongest inhibition was observed when NQO1-positive cells were co-treated with Gem and 5’-DFUR, supporting our clinical data from ESPAC.

**Conclusion:**

High tumor NQO1 predicts better outcome following GemCap therapy.

## Introduction

PDAC is a leading cause of cancer-related deaths, with a 5-year survival rate of ∼12%.^1^ Patients eligible for potentially curative resection have significantly better outcomes.^2-5^ ESPAC-4, which randomized resected patients to adjuvant gemcitabine (Gem) or gemcitabine plus capecitabine (GemCap), was the first practice-changing combination trial in this setting.^6^ GemCap significantly improved median overall survival (28.0 months; 95% CI = 23.5 to 31.5) compared with Gem (25.5 months; 95% CI = 22.7 to 27.9), although the basis for the improvement is not well understood. Capecitabine, an orally available prodrug, is sequentially metabolized to its active form 5-fluorouracil (5-FU).^7^ A prior ESPAC trial, ESPAC-3[V2], demonstrated equivalent survival for patients randomized to 5-FU plus folinic acid or Gem.^4^

**Figure 1.**
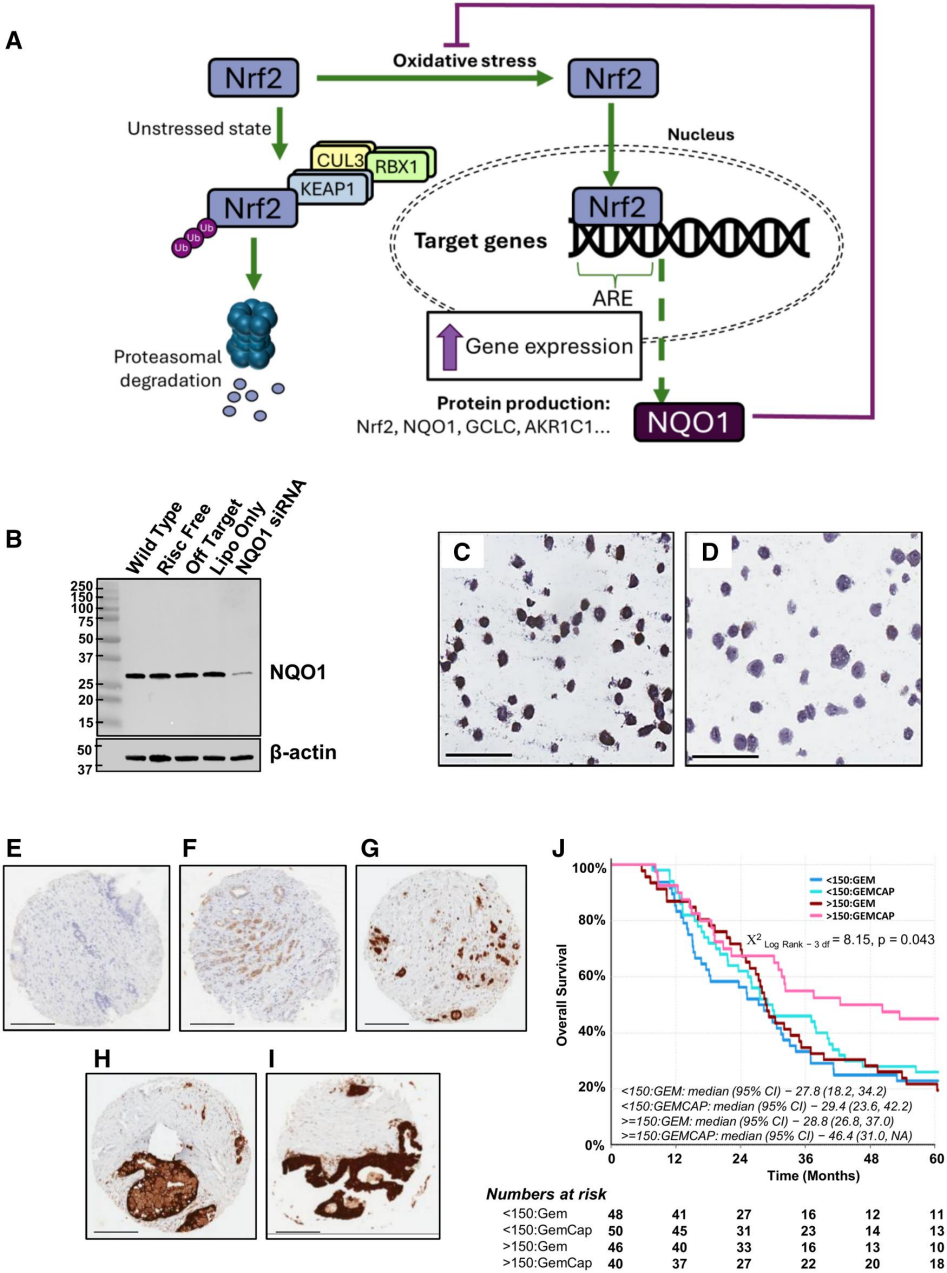
The Nrf2/NQO1 axis, immunohistochemical analysis of NQO1 levels, and survival. **(A)** Illustration of the Nrf2 activation pathway. During periods of low oxidative stress, Nrf2 is ubiquitinated by Keap1/RBX1/Cul3 and degraded via the proteasome. Oxidative stress disrupts this mechanism and allows Nrf2 to accumulate, translocate to the nucleus, and bind to the antioxidant response element (ARE) in the promoter regions of target genes such as NQO1, activating their transcription. A negative feedback loop restores function of Keap1-mediated degradation. **(B-D)** Assessment of antibody specificity for immunocytochemistry. Western blot image of MIA PaCa-2 whole cell lysates **(B)** showing antibody specificity following NQO1 depletion by siRNA. MIA PaCa-2 cells treated with control **(C)**, or NQO1-targeting siRNA (20 nM) **(D)** were formalin fixed and embedded in paraffin and stained for NQO1. Scale bar = 100 µm. **(E-I)** NQO1 immunohistochemistry scoring. Representative images of NQO1 negative **(E)**, blush **(F)**, mild **(G)**, moderate **(H)**, and strong **(I)** expressing tumors. Scale bar = 200 µm. **(J)** Kaplan Meier plot showing overall survival of ESPAC patients when dichotomized into high and low NQO1 protein levels at an H-score cut-off of 150, when treated with Gem or GemCap. Abbreviations: Nrf2 = nuclear factor erythroid 2–related factor 2; NQO1 = NAD(P)H quinone dehydrogenase 1; GCLC = glutamate–cysteine ligase catalytic subunit; AKR1C1 = Aldo-keto reductase family 1 member C1; Cul3 = Cullin 3; RBX1 = Ring-box 1; Ub = Ubiquitin; Keap1 = Kelch-like ECH-associated protein 1; ARE = antioxidant response element.

NAD(P)H quinone dehydrogenase 1 (NQO1), an antioxidant enzyme overexpressed in PDAC^8-10^ catalyzes the 2-electron reduction of quinones,^11^ preventing reactive oxygen species formation. NQO1 is a downstream target of Nuclear factor erythroid 2-Related Factor 2 (Nrf2), a principal modulator of the cellular antioxidant response.^12^ In the absence of cellular stress, Nrf2 levels are suppressed (Figure 1, A).^13^ In response to cytotoxic stress, Nrf2 accumulates and activates target genes with antioxidant response elements (AREs), such as NQO1.^13^ NQO1 is also influenced by a biologically significant germline single-nucleotide polymorphism (SNP) C609T, which results in a proline-to-serine amino-acid substitution at codon 187 (NQO1*2; rs1800566) reducing protein stability and level.^14^^,^^15^ Heterozygous (CT) cells express lower NQO1 protein levels than those homozygous for the wild-type allele (CC).^15^

We hypothesized that high tumoral NQO1 would confer chemotherapeutic resistance, leading to poor patient outcome. Unexpectedly, we found, using archival material from ESPAC-4,^6^ that high NQO1 levels were associated with better survival in GemCap-treated patients (but not in those treated with Gem monotherapy), suggesting that NQO1 may serve as a predictive biomarker for PDAC treatment. Since the C609T SNP, measurable by a blood test, would make a more translatable biomarker, it was also assessed.

Finally, NQO1 tumor data represent pre-treatment baseline levels while survival outcomes were assessed following post-surgical chemotherapy (targeting microscopic residual disease). Understanding whether Nrf-2/NQO1 was activated during postsurgical chemotherapy could explain why high NQO1 predicted better outcome in GemCap-treated patients. We reasoned that strong Nrf2 pathway activation might, by upregulating phase II detoxification enzymes, enhance treatment tolerability and thereby improve outcomes. Our investigations, however, revealed limited Nrf2/NQO1 induction following drug exposure (in either Nrf2 reporter mice or PDAC cell lines); instead, high drug concentrations down-regulated this cytoprotective axis in cell lines. However, NQO1 depletion and colony-forming assays revealed a role for NQO1 in suppressing tumor growth and/or dissemination. This inhibitory effect was most pronounced in NQO1-positive cells treated with combination Gem and 5’-DFUR (a capecitabine metabolite), aligning with our observations from the ESPAC trial.

## Materials and methods

### Ethics statement

Ethical approval for the ESPAC-4 trial was from the Liverpool Adult Research Ethics Committee (March 4, 2008) and obtained in all other participating countries. Patients gave written informed consent. Sample analysis was conducted under a Health Research Authority approved study (11/NW/0083 and 16/LO/1630).

### NQO1 measurements in ESPAC specimens

For immunostaining, following antigen retrieval and washing steps, sections (4 µm and 5 µm, respectively) from formalin-fixed paraffin-embedded (FFPE) ESPAC tumor tissue microarrays (TMAs) or paraffin-embedded MIA PaCa-2 cell pellets were stained for NQO1 (Monoclonal Antibody; ThermoFisher Scientific, Invitrogen MA1-16672) and scored by specialist histopathologist FC, blinded to treatment arm/patient outcomes. Full details of immunostaining and germline NQO1 SNP analysis can be found in the Supplementary Material.

### In vivo and in vitro assays

Gemcitabine hydrochloride (Sigma-Aldrich, G6423) and 5-FU (Sigma-Aldrich, F6627) were dissolved in H_2_O, CDDO-Me (Sigma-Aldrich) in DMSO and 5’-DFUR (Sigma-Aldrich, F8791) in H_2_O or DMSO, as indicated. Experiments using Nrf2 reporter mice, cell lines, protein knockdown, Western analysis, Nrf2 luciferase assay, and colony formation/quantification are provided in Supplementary Material.

### Statistical methodology

Continuous ESPAC data were summarized as median (IQR) and categorical data as frequencies of counts with associated percentages. The outcome of interest was overall survival measured as the time from randomization until death by any cause. For overall summaries and the comparison of categorical covariates, estimates of overall survival were obtained using the Kaplan and Meier method. For continuous data, the impact of overall survival was evaluated using weighted Kernel Estimators. Further details can be found in the Supplementary Material.

## Results

### NQO1 tumoral levels and survival

Using a mouse monoclonal NQO1 antibody (MA1-16672), a 31 kDa band, reduced in intensity by NQO1-targeting siRNA, was detected (MIA PaCa-2; Figure 1, B). Immunocytochemical staining of paraffin-embedded MIA PaCa-2 cells revealed diminished staining in NQO1-depleted cells (Figure 1, D vs Figure 1, C). This antibody was used for immunohistochemical staining of pre-treatment tumor tissue from ESPAC-4^6^ (Figure 1, E-I). Of 732 ESPAC-4 patients, clinical data were extracted from 184 (comprising 142 [77%] deaths) for which tissue was available for NQO1 protein level assessment. Clinical characteristics, including overall survival, comparing 184 patients included versus those omitted are in Table S1 and Figure S1. For those included, median overall survival was 28.5 months (IQR = 25.2-33.11) for Gem and 34.69 months (IQR = 27.6-46.62) for GemCap (Table 1). Univariable analyses identified tumor differentiation, maximum tumor size, post-operative CA19-9, lymph node positivity, and adjuvant therapy as predictive of outcome (*P* < .05).

**Table 1.** Summary of censor events, median survival times, and univariable and multivariable Cox proportional hazards model outputs.

Variable	Category	Number of events (%)	24-month survival rate (IQR)	Univariable Cox models		Multivariable Cox model	
				Hazard ratio (95% CI)	*P*	Hazard ratio (95% CI)	*P*
Sex							
	Female	96 (75)	27.99 (22.27-32.1)				
	Male	88 (67)	33.74 (28.65-41.26)	0.85 (0.61 to 1.18)	.341		
Smoking status							
	No	67 (48)	30.68 (27.33-49.38)				
	Past	57 (41)	34.23 (26.05-52.96)	0.98 (0.65 to 1.49)			
	Present	24 (21)	26.26 (18.59-40.08)	1.44 (0.86 to 2.41)	.122		
	Missing	31 (21.7)					
Diabetic status							
	No	23 (17)	25.2 (18.13-NA)				
	Not insulin dependent	114 (88)	30.53 (27.53-40.08)	0.98 (0.58 to 1.64)			
	Insulin dependent	17 (12)	31.87 (16.07-NA)	0.91 (0.43 to 1.91)	.795		
WHO performance status							
	0	82 (67)	29.42 (24.97-35.22)				
	1	99 (73)	30.68 (27.6-41.19)	0.84 (0.6 to 1.17)			
	2	3 (2)	15.54 (8.58-NA)	1.04 (0.25 to 4.23)	.29		
Tumor difference							
	1	18 (15)	35.92 (29.93-61.7)				
	2	118 (86)	32.08 (27.93-42.25)	0.95 (0.55 to 1.64)		0.91 (0.524 to 1.59)	
	3	48 (41)	22.65 (17.61-30.19)	1.53 (0.85 to 2.77)	.01**	1.4 (0.771 to 2.546)	.11
Maximum tumor size							
	<30	110 (83)	31.08 (27.6-40.08)				
	≥30	73 (58)	29.24 (25.36-36.99)	1.14 (0.81 to 1.59)	.45		
	Continuous			1.2 (1.024 to 1.397)	.024**		
Postoperative CA 19-9				1.001 (1 to 1.001)	<.001**		
	<180	142 (106)	31.97 (28.65-40.37)				
	≥180	24 (21)	17.41 (11.7-29.24)	2.23 (1.39 to 3.57)	.001**		
	Continuous			1.26 (1.113 to 1.425)	<.001**	1.26 (1.105 to 1.436)	<.001**
Lymph node							
	Negative	153 (124)	28.65 (25.69-31.6)				
	Positive	31 (18)	65.14 (37.09-NA)	0.49 (0.3 to 0.81)	.004**	0.63 (0.377 to 1.052)	.002**
Resection margin							
	Negative	118 (95)	29.09 (25.92-32.29)				
	Positive	66 (47)	35.91 (26.81-61.7)	0.76 (0.54 to 1.08)	.125		
NQO1 genotype							
	CC	125 (95)	30.68 (27.93-41.06)				
	CT	54 (45)	27.56 (23.59-34.23)	1.31 (0.92 to 1.87)			
	TT	2 (2)	40.13 (15.24-NA)	1.34 (0.33 to 5.44)	.128		
NQO1 H-score							
	<150	92 (74)	28.63 (25.2-36.96)				
	≥150	92 (68)	32.08 (28.35-49.38)	0.78 (0.56 to 1.09)	.145		
	Continuous			0.91 (0.812 to 1.028)	.135		
Treatment							
	Gemcitabine	94 (80)	28.5 (25.2-33.11)				
	GEMCAP	90 (62)	34.69 (27.6-46.62)	0.67 (0.48 to 0.94)	.018**	1.15 (0.325 to 4.082)	.056

The number of censor events is presented for different categories of categorical variables and for continuous variables for which there are missing data, alongside the percentage of overall events for a given variable in parentheses. Medians are presented for the same variables and categories, with interquartile ranges in parentheses. Hazard ratios, with 95% confidence intervals in parentheses, along with *P*-values, are presented for categorical variables for comparator groups in models. To produce meaningful hazard ratios, all continuous variables (save Post-Operative CA 19-9 due to issues of non-proportionality when transformed) were transformed. Maximum tumor size was square root transformed. Significant *P*-values are denoted by **.

Dichotomizing groups into high and low NQO1 expressors at an H-score cut-off of 150 permitted a relatively even distribution of patients per group. Kaplan-Meier analyses gave median survival estimates of 46.4 months (IQR = 31.01-NA) for high NQO1 expressors versus 29.4 months (IQR = 23.6-42.2) for low expressors in the GemCap-group (Figure 1, J).

A multivariable model was constructed that included NQO1 as a nested covariate within treatment arm. Tumor differentiation, lymph node status, and post-operative CA19-9 were selected as independent indicators of overall survival, while treatment allocation was the main covariate of interest. NQO1 H-score was included both as a main effect (to assess prognostic value) and as an effect nested within treatment allocation (to assess the predictive value). The best-performing model included NQO1 H-score as a nested (predictive) effect (Table 2). A high NQO1 H-score was associated with a decreased risk of death in the GemCap group (HR = 0.87 [95% CI = 0.751 to 0.999]; *P* = .049) but had no measurable effect in the Gem group (HR = 0.98 [95% CI = 0.78 to 1.23]; *P* = .867).

**Table 2.** Summary of censor events, median survival times, and univariable and multivariable Cox proportional hazards model outputs for NQO1 treatment by H-score and genotype.

Variable	Category	Number of events (%)	24-month survival rate (IQR)	Univariable Cox models		Multivariable Cox model	
				Hazard ratio (95% CI)	*P*	Hazard ratio (95% CI)	*P*
Treatment by NQO1 H-score							
Gemcitabine							
	<150	49 (42)	27.33 (18.17-34.23)				
	≥150	45 (38)	28.88 (25.69-39.42)	0.89 (0.58 to 1.38)	.612		
	Continuous			1.01 (0.814 to 1.253)	.93	0.98 (0.784 to 1.227)	.867
GEMCAP							
	<150	51 (38)	29.93 (25.36-42.25)				
	≥150	39 (24)	42.45 (31.01-NA)	0.71 (0.43 to 1.19)	.193	One	
	Continuous			0.86 (0.742 to 0.991)	.037**	0.87 (0.751 to 0.999)	.049**
							
Treatment by NQO1 genotype							
Gemcitabine							
	CC	66 (56)	28.12 (24.24-36.96)				
	CT	25 (22)	29.24 (18.4-35.22)	1.14 (0.7 to 1.88)	.611		
GEMCAP							
	CC	59 (39)	41.06 (30.19-82.29)				
	CT	29 (23)	26.05 (21.65-37.75)	1.53 (0.91 to 2.57)	.109		

The number of censor events is presented for different categories of categorical variables and for continuous variables for which there are missing data, alongside the percentage of overall events for a given variable in parentheses. Medians are presented for the same variables and categories, with interquartile ranges in parentheses. Hazard ratios, with 95% confidence intervals in parentheses, along with *P*-values, are presented for categorical variables for comparator groups in models. To produce meaningful hazard ratios, continuous variables were transformed. NQO1 H-score was transformed by the equation log(*x*)+1. Significant *P*-values are denoted by **

### NQO1 C609T SNP and survival

**Figure 2.**
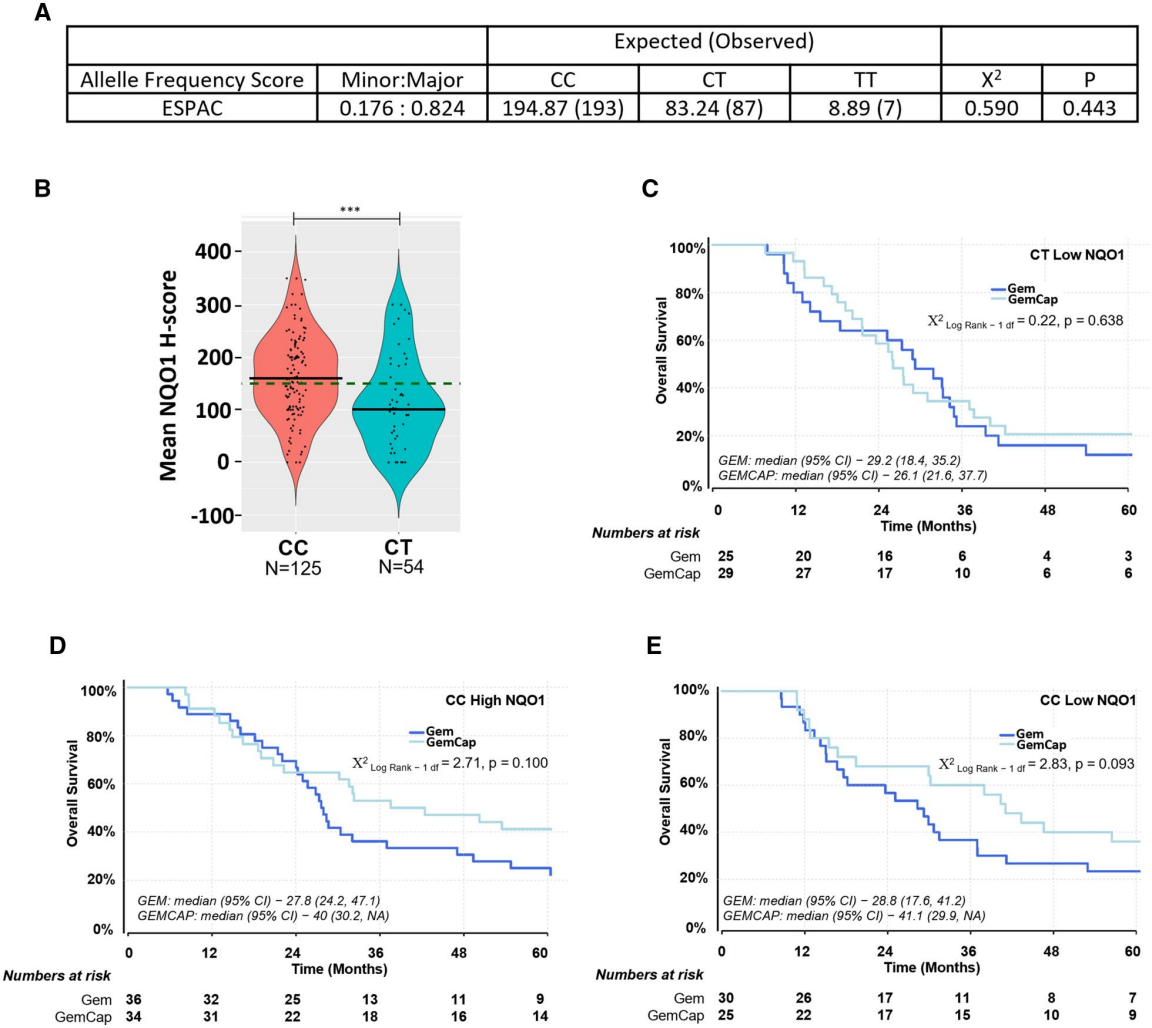
Germline single-nucleotide polymorphism C609T status versus protein level and outcome. **(A)** Genotype frequencies were as expected based upon Hardy-Weinberg equilibrium and comparison with the European gnomAD v2.1.1 cohort (*P* = .443). **(B)** Violin Plots of the distribution of NQO1 H-score in patients homozygous for the stable variant of NQO1 compared to those with the CT germline status, Wilcox test (***P < .001). **(C-E)** Kaplan Meier plots showing survival of ESPAC patients with the CC or CT genotype and high (above 150 H-score) or low (below 150 H-score) NQO1 levels.

We next assessed whether germline NQO1 C609T SNP status correlated to tumoral NQO1 protein levels and could predict response to GemCap. Samples from 287 patients from ESPAC-1/3 or ESPAC 4 were available for genotyping, of which 67.2% had the CC genotype, 30.3% CT, and 2.4% TT. These frequencies were in Hardy-Weinberg equilibrium with the European gnomAD v2.1.1 cohort^16^ (Figure 2, A), indicating no evidence of increased risk of PDAC associated with any variant.

Further analyses focused on ESPAC-4 patients with matched SNP and NQO1 expression data. In patients with the rare TT genotype (*n* = 2), NQO1 protein was undetectable (H-score = 0). Due to low number, these were excluded from subsequent analyses. The median NQO1 H-score was significantly higher in patients homozygous for the stable CC variant (*n* = 125; 160 [IQR = 103.75–217.50]) compared to CT patients (*n* = 54; 100.83 [IQR = 51.56–177.92]; *P* < .001; Figure 2, B). Median survival was 30.68 (IQR = 27.93–41.06) months and 27.56 (IQR = 23.59–34.23) months, respectively, for CC and CT genotypes (*P* = .128): clinical characteristics of CC and CT groups are compared in Table S2.

Most CT patients (38/54; 70%) were low NQO1 expressors (H-score of <150). For this group, there was no discernible difference in survival between Gem or GemCap treatment (*P* = .638; Figure 2, C). Too few high NQO1 expressors with the CT genotype (*n* = 16) were available to analyze. By contrast, the majority of CC patients were high NQO1 expressors (70/125; 56%). Trends towards better outcome following GemCap compared to Gem in CC patients whether NQO1 was high or low were not statistically significant (Figure 2, D and E; Table 2) and were therefore excluded from multivariable analysis. In summary, high NQO1 H-score (not C609T status) predicted risk of death for patients treated with GemCap.

### Effects of systemic gem or 5-FU on Nrf2 induction

**Figure 3.**
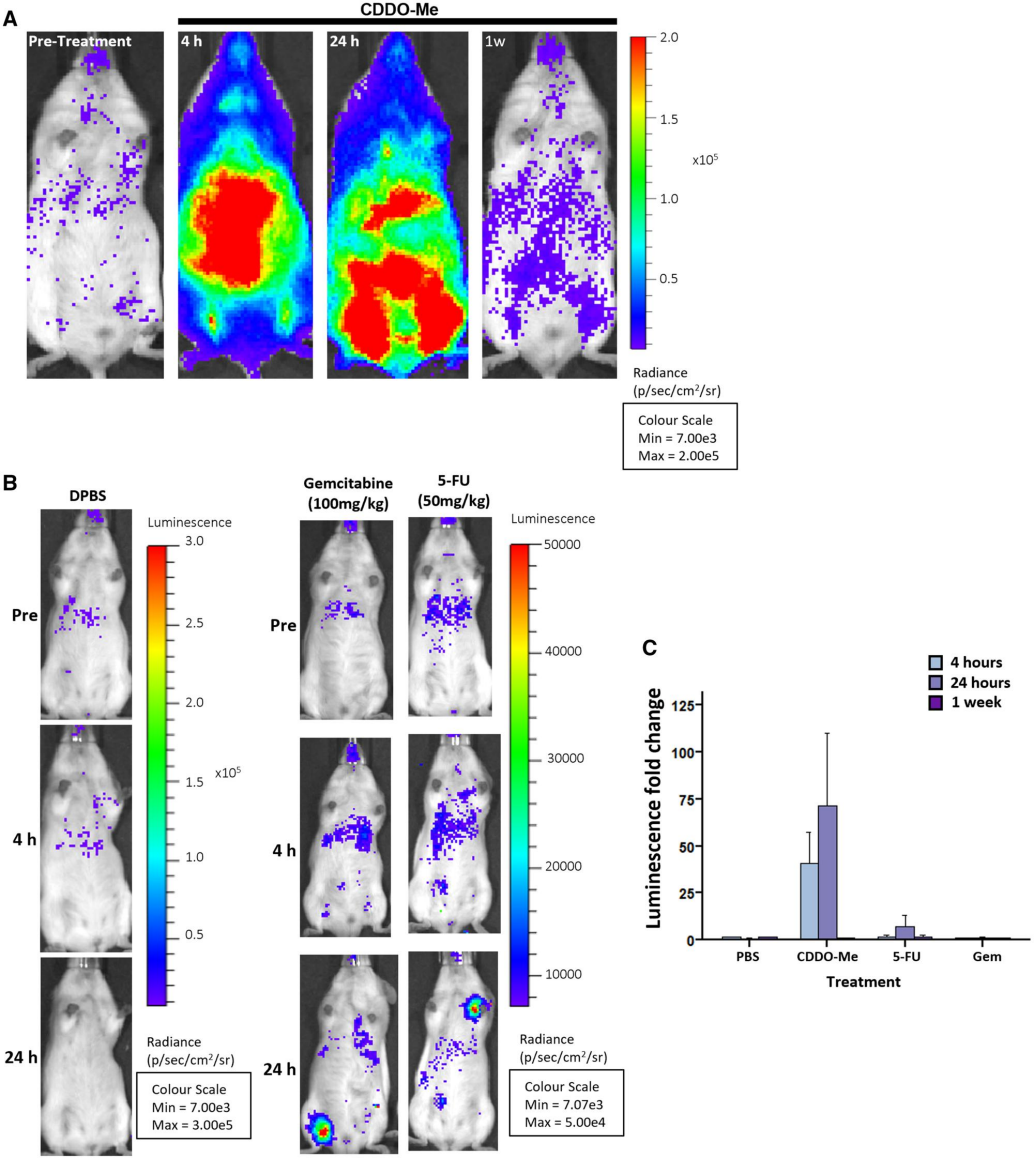
Gemcitabine or 5-FU treatment of Nrf2-reporter mice. **(A)** Representative image of a single OKD48+/− mouse prior to treatment with CDDO-Me and at 4 h, 24 h, and 1 week (1w) post-treatment (10 mg/kg). The location of intensity of radiance, as a marker of Nrf2 activity, is represented by colored overlay. **(B)** Representative images of mice treated with vehicle (DPBS) only, gemcitabine (100 mg/kg), or 5-FU (50 mg/kg). Small areas of localized Nrf2 activation were detected irregularly in all treatment arms as seen at 24 h. From a total of 16 mice treated with Gem, 2 mice (at 24 h post-treatment with 0.5 mg/kg and 50 mg/kg, respectively) had luminescence localized adjacent to a limb. A total of 30 mice were treated with different concentrations of 5-FU, of which 2 of 14 mice (treated with 50 mg/kg 5-FU) showed localized Nrf2 induction. **(C)** Mean of the average luminescence readings from CDDO-Me, gemcitabine (100 mg/kg), or 5-FU (50 mg/kg)-treated mice at 4 h, 24 h, and 1 week (1w) normalized to pre-treatment readings. Errors bars + SEM. ***P < .001.

Considering that (1) NQO1 is inducible via the Nrf2-mediated response to electrophilic and oxidative stress^17^ and (2) GemCap-treated patients experienced more grade 3-4 adverse events than those on Gem alone in ESPAC-4,^6^ we questioned whether the superior outcome of GemCap-treated high NQO1 expressors related to enhanced capacity to tolerate treatment. To assess systemic Nrf2 activation by Gem or 5-FU (the metabolite of capecitabine), we used cancer-free OKD48 transgenic mice, which respond to oxidative stress in a Keap1-Nrf2-dependent manner by eliciting a luminescence signal.^18^ Exposure to the pharmacological Nrf2 activator CDDO-Me significantly increased luciferase activity, which was observed at 4 hours post-treatment, remained elevated at 24 hours, and returned toward baseline levels by 1 week post-treatment (Figure 3, A and C). By contrast, Gem or 5-FU treatment did not induce luminescence (Figure 3, B and C), apart from occasional small, localized signals adjacent to limbs (see Figure 3, B), observed in individual mice. Thus, Nrf2 was not strongly induced following systemic Gem or 5-FU treatment.

Grade 3-4 adverse events were more frequent in patients randomized to GemCap than Gem (53.9% vs 46.1%; *P* = .01; Table S3^6^). However, C609T germline status was not associated with adverse events, occurring in 112/186 (60%) of CC patients and 43/81 (53%) of CT (*P* = .278) (Table S3). Tumoral NQO1 H-score was also not correlated with adverse events (Table S3). Taken together our data suggested that the ability to produce stable NQO1 (CC genotype) was not protective against the adverse effects of chemotherapy.

### Effects of chemotherapy on Nrf2/NQO1 levels in PDAC cells

**Figure 4.**
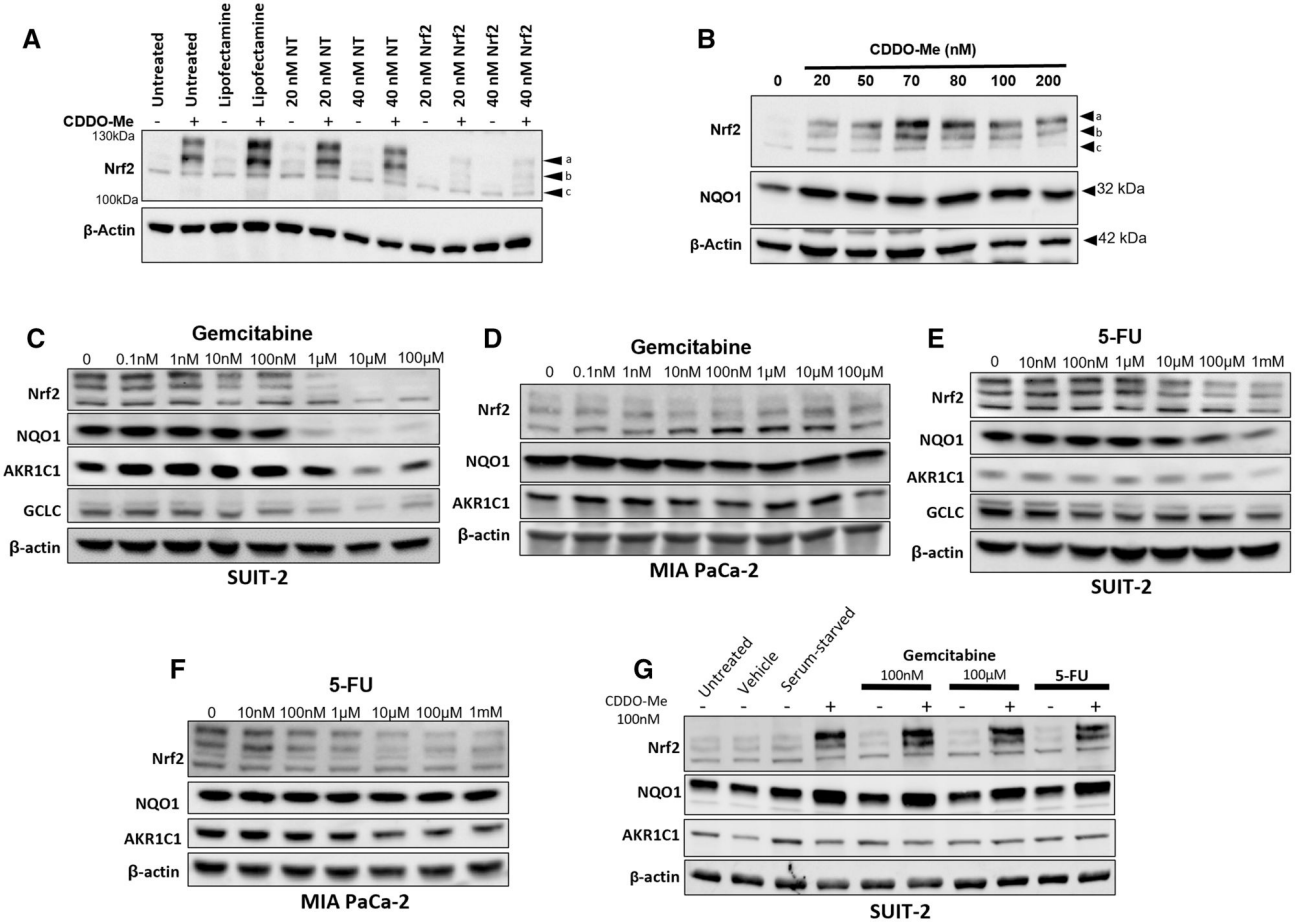
Nrf2 and downstream protein levels following gemcitabine and 5-FU treatment of PDAC cell lines. **(A)** Western blot of SUIT-2 cell lysates 24 h post-treatment with non-targeting (NT) and Nrf2-targeting (Nrf2) siRNAs +/− exposure to 100 nM CDDO-Me. **(B)** Representative image (from three independent experiments) of SUIT-2 cell lysates, following 24 h exposure to indicated concentrations of CDDO-Me, probed for Nrf2 and downstream target NQO1. **(C-F)** Western blots of Nrf2 and downstream proteins from SUIT-2 (**C** [*n* = 3] and **E** [*n* = 3]) and MIA PaCa-2 (**D** [*n* = 3] and **F** [*n* = 1]) following 24 h of treatment with gemcitabine (**C** and **D**) or 5-FU (**E** and **F**), respectively. Following 24 h treatment with 1 µM, 10 µM, and 100 µM Gem (**C**), SUIT-2 cell lysates contained lower levels of Nrf2, accompanied by a corresponding loss of NQO1, and other downstream proteins, including aldo-keto reductase 1C1/2 (AKR1C1/2) and glutamate-cysteine ligase catalytic subunit (GCLC). In MIA PaCa-2 cells (**D**) NQO1 levels were diminished in intensity following treatment with concentrations of 100 nM Gem and above. 5-FU treatment (**E, F**) also caused a decrease in Nrf2 at higher concentrations (from 10 µM in SUIT-2 cells and 100 nM in MIA PaCa-2 cells). In SUIT-2 cells, NQO1 and GCLC levels progressively diminished following treatment at the higher concentrations of 5-FU. NQO1 levels were not affected in MIA PaCa-2 cells treated with 5-FU. **(G)** Western blot of Nrf2 and downstream proteins from SUIT-2 cells treated with gemcitabine (100 nM or 100 µM), or 5-FU (100 µM) +/− CDDO-ME or as control, vehicle treated for 24 h or cultured in FBS-free medium (“serum-starved”) for 24 h prior to harvest (*n* = 1). β-Actin levels were ascertained as a control for loading.

We next explored whether post-surgical chemotherapy could influence NQO1 tumoral levels via the Nrf2-mediated adaptive response (Figure 4; Figure S2). Two distinct Nrf2-antibody-reactive species between 100 kDa and 130 kDa (a and b, Figure 4, A), but not a third species (designated c) increased following treatment with CDDO-Me (Figure 4, A and B) or decreased following knockdown of Nrf2 (Figure 4, A). By contrast, exposure of SUIT-2 (TT genotype) and MIA PaCa-2 (CT genotype) cells to Gem or 5-FU resulted in stable, or at higher drug concentrations, reduced Nrf2 and downstream protein levels (Figure 4, C-F). Note that broad concentration ranges of Gem and 5-FU were used, which span peak plasma concentrations for Gem^19^ and target plasma concentrations for 5-FU.^20^

Concerned that chemotherapeutic drug treatment may have rendered cells incapable of inducing Nrf2, we co-treated SUIT-2 cells with high concentrations of Gem or 5-FU and the Nrf2 inducer CDDO-Me. Cells remained capable of synthesizing and accumulating Nrf2 and NQO1 (Figure 4, G). Similarly, under conditions of serum starvation, cancer cells maintained Nrf2 levels. We concluded that although Nrf2 induction was possible in Gem- or 5-FU-treated cells, the cellular response was rather for Nrf2 to remain unchanged or, at higher drug concentrations, to incur reductions in its protein levels.

To determine Nrf2 transcriptional activation following Gem or 5-FU treatment, an Nrf2-inducible plasmid containing 8 X Nrf2-responsive ARE sequences upstream of luciferase was used (Figure S3, A and B). CDDO-Me increased luciferase activity. Exposure of MIA PaCa-2 cells to 100 µM Gem resulted in a 3.4-fold increase in Nrf2 activity (*P* = .002). Neither PANC-1 nor SUIT-2 cells exhibited significant increases in Nrf2 activity following Gem treatment. Modest increases in Nrf2 activity following 5-FU treatment did not reach statistical significance in any of the cell lines tested.

### Effects of 5’-DFUR on NQO1 protein levels

**Figure 5.**
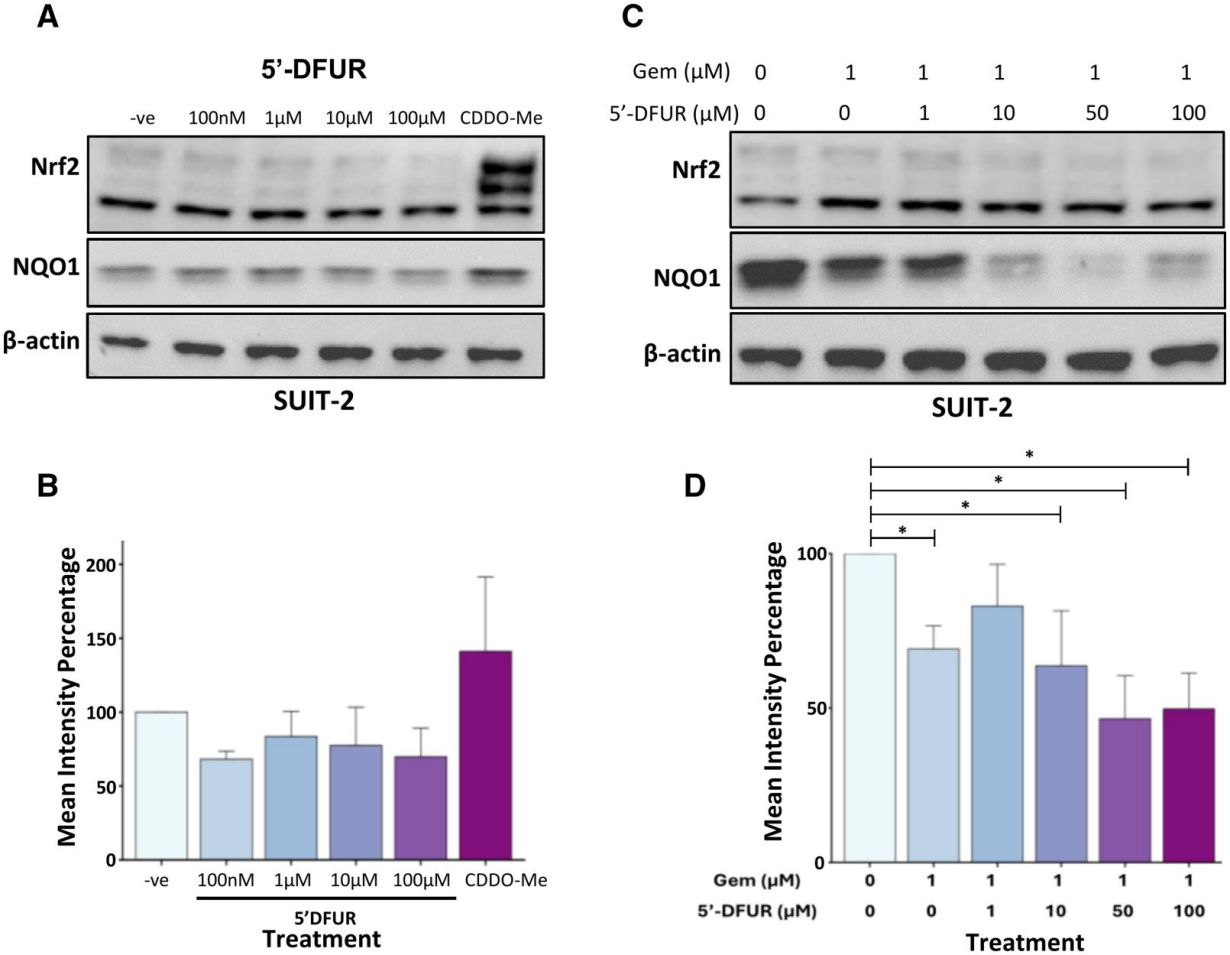
NQO1 protein levels following 24 h treatment with gemcitabine and 5’-DFUR. **(A)** Representative Western blot images showing the expression of indicated proteins in SUIT-2 cell lysates 24 hours post-treatment with increasing concentrations of 5’-DFUR. The Nrf2 inducer CDDO-Me was included as a positive control, while vehicle-only treatment served as a negative control (−ve) (*n* = 3). β-Actin was used as a loading control. **(B)** Quantification of NQO1 band intensity for SUIT-2 normalized to β-actin and expressed as a percentage of the negative control. **(C)** Western blot images of SUIT-2 (*n* = 4) cell lysates treated with increasing concentrations of 5’-DFUR, in combination with a fixed concentration of gemcitabine (1 µM). **(D)** Quantification of NQO1 band intensity for SUIT-2 normalized to β-actin and expressed as a percentage of the negative control. Significant differences relative to the negative control were determined via Mann-Whitney *U* tests with Benjamini-Hochberg multiple comparison corrections. Error bars + SEM. *P < .05.

While 5-FU did not induce NQO1, we questioned whether 5’-DFUR, an intermediate capecitabine-metabolite^7^ could. Treatment of SUIT-2 cells with 5′-DFUR for 24 hours (Figure 5, A and B) did not result in significant alterations in NQO1 levels. In PANC-1 cells (Figure S4, A and C), NQO1 expression tended to increase modestly with 5’-DFUR treatment, while the opposite trend was observed in MIA PaCa-2 cells (Figure S4, B and D). Treating PDAC cells with combined 5’-DFUR and Gem did not mitigate Gem-induced downregulation of NQO1. In SUIT-2 cells, the significant reduction in NQO1 levels observed with 1 µM Gem (*P* = .026; Figure 5, C and D) was modestly disrupted at low levels of 5’-DFUR, but was sustained when cells were co-treated with 1 µM Gem plus 10 µM (*P* = .026), 50 µM (*P* = .026), or 100 µM (*P* = .026) 5’-DFUR. Treating MIA PaCa-2 cells (CT, with higher NQO1 baseline levels than SUIT-2) with 1 µM Gem plus increasing concentrations of 5’-DFUR did not significantly alter NQO1 levels (Figure S4, E and F).

### Functional analysis of NQO1 in PDAC cells

**Figure 6.**
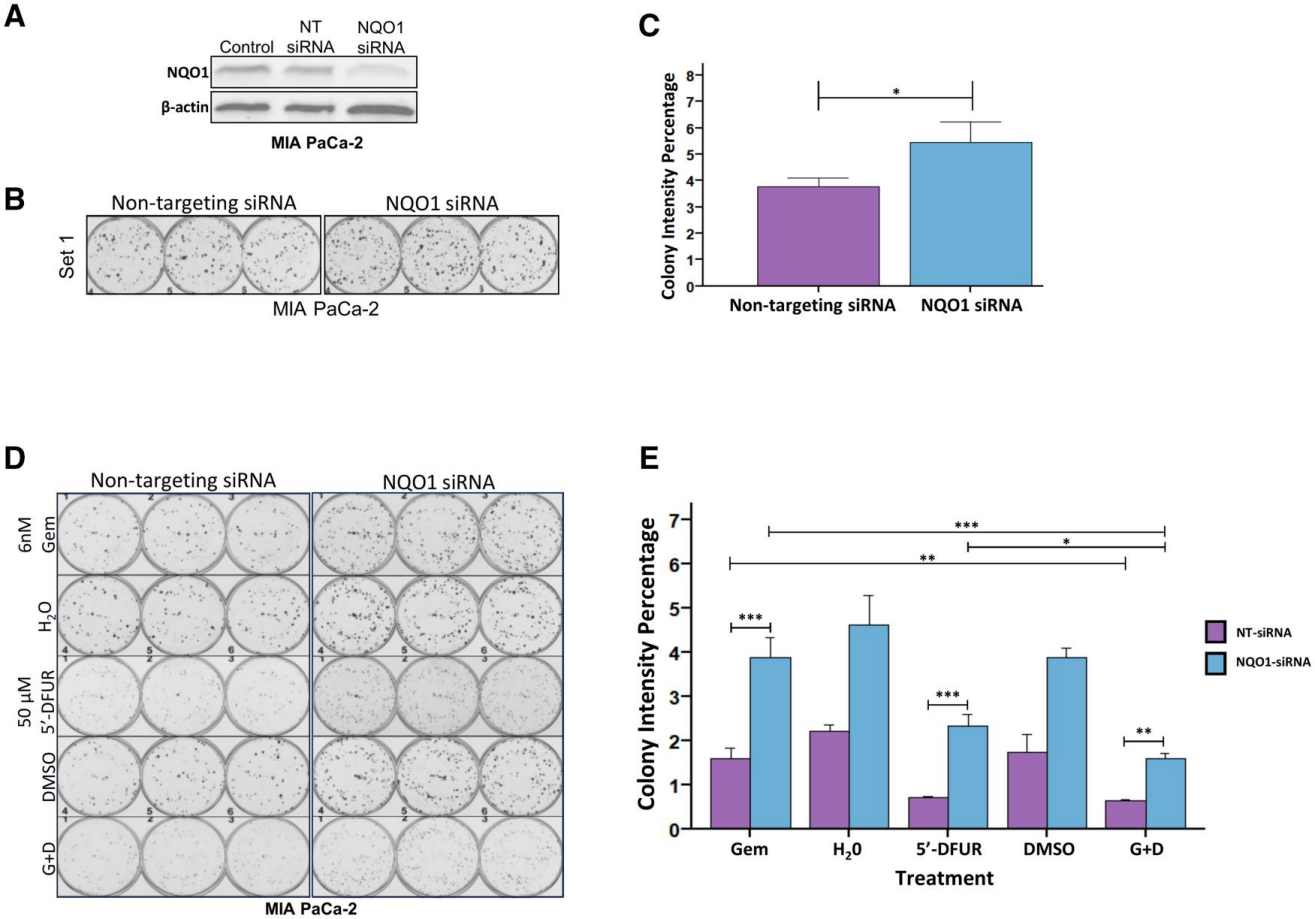
Effects of NQO1 knockdown and drug treatments on colony formation. **(A)** Western blot analysis of MIA PaCa-2 lysates 48 h post-treatment with non-targeting or NQO1-targeting siRNA. **(B)** Evaluation of the effect of NQO1 knockdown on colony formation in MIA PaCa-2 cells (*n* = 2) after 24 h incubation with non-targeting or NQO1-targeting siRNA, re-seeding and incubation up to 10 days. **(C)** Column chart of Average Colony Intensity Percentage of MIA PaCa-2 cells (from **B**). **(D-E)** Evaluation of the effect of indicated drugs combined with NQO1 knockdown on colony formation in MIA PaCa-2 cells (seeded at 500 cells/well) (**D**) with Average Colony Intensity Percentages data presented (**E**). H_2_O and DMSO are respective vehicle controls for gemcitabine and 5’-DFUR. G+D = combined gemcitabine and 5’-DFUR. Two-way ANOVA with Tukey’s post-hoc test was used to assess inter-group and inter-treatment differences (vehicle controls excluded). Error bars + SEM. *P < .05, **P < .01, ***P < .001.

We next tested the effects of NQO1 depletion and individual or combined drug treatments on colony formation (Figure 6). Strikingly, siRNA-mediated NQO1 depletion from MIA PaCa-2 cells increased the average colony intensity percentage (a measure which combines both the number of colonies and their staining intensity/size^21^; Figure 6, A-C; *P* = .026, Figure S5, A and B; *P* = .046), indicating that NQO1 suppresses colony formation. Treatment with Gem or 5’-DFUR also impaired colony formation compared to vehicle controls; the most effective impediment to colony formation was the combination of Gem and 5’-DFUR in cells containing NQO1 (Figure 6, D and E). This effect was also observed when MIA PaCa-2 cells were seeded at a lower density and treated with a lower concentration of 5’-DFUR (Figure S5, C and D) and was similarly observed in SUIT-2 cells (Figure S6, A-C). Colony number and size/diameter data are presented separately (Figure S6, D-G). Both were significantly reduced following combined Gem and 5’-DFUR treatment, the effect more pronounced on colony number.

## Discussion

We report that elevated pre-treatment tumoral NQO1 protein levels predicted longer survival in ESPAC-4 patients randomized to GemCap but not to Gem monotherapy. This result was initially unexpected, given NQO1’s reported role in chemo-resistance^22^^,^^23^ and its previous association with poor PDAC survival.^10^ However, to our knowledge, this is the first study correlating NQO1 expression with post-chemotherapy outcomes in PDAC. Bias was minimized by using patient tissue from the ESPAC-4 randomized controlled trial, where sample collection was standardized, clinical data externally monitored and quality controlled, and NQO1 analyses undertaken blinded to all data, including treatment arm. Moreover, rigorous antibody validation was corroborated by genotype data, with NQO1 undetectable in patients homozygous for the unstable mutant NQO1*2 protein (TT), and significantly higher in the CC than in the CT patient group.

Given that the NQO1 C609T polymorphism, measurable via a blood sample, would make a more practical biomarker, evaluating its potential to predict response to GemCap was a logical next step. Despite a good correlation, C609T SNP status did not improve the predictive performance of NQO1 tumor levels. NQO1 is highly inducible,^17^ which limits the extent to which SNP status alone governs its expression. Consistent with this, we observed considerable variability in H-score among both CC and TT patients. Nonetheless, important patterns emerged. We noted that patients with the CT genotype were mostly (70%) low NQO1 expressors, and among this CT/low-NQO1 subgroup, there was no discernible difference in survival benefit between Gem and GemCap. CC patients had more variable H-scores, though 56% were high NQO1 expressors. Trends towards better outcome following GemCap versus Gem did not achieve statistical significance. Finally, although the NQO1 C609T polymorphism has been associated with increased risk of other cancers,^24-26^ our genotyping of 287 patients enrolled in ESPAC-1/3 or ESPAC 4 did not support it as a risk factor for resectable PDAC.

Pancreatic cancer is a systemic disease, and adjuvant chemotherapy targets microscopic, disseminated tumor cells. However, many patients are unable to complete treatment due to chemotherapy-related toxicity and adverse events,^27^ prompting us to investigate whether elevated NQO1 levels signify an enhanced capacity for Nrf2 activation and, consequently, improved detoxification. Unlike CDDO-Me, or other agents we tested in Nrf2-responsive OKD48 mice,^28^ systemic treatment with Gem or 5-FU did not induce Nrf2 activation. Moreover, the occurrence of adverse side effects was independent of NQO1 C609G status or tumoral levels of NQO1, indicating that elevated NQO1 levels do not protect from the side-effects of chemotherapy. These findings led us to focus attention on properties intrinsic to tumor cells themselves that might underpin the observed correlation between high tumoral NQO1 expression and treatment response.

Treatment of cultured pancreatic cancer cells with Gem or 5-FU caused NQO1 to remain unchanged or, at higher drug concentrations, to incur reductions in its protein levels. The downregulation of NQO1 was more profound in SUIT-2 (TT) cells, which express low baseline levels of NQO1, compared to MIA PaCa-2 (CT) cells, which have higher NQO1 expression. To investigate a functional role for NQO1 in PDAC cells, colony formation assays were performed. These revealed that NQO1 suppresses colony growth, its depletion leading to increased colony formation. The suppressive effect of NQO1 on colony formation persisted in the presence of individual chemotherapeutic drugs, but was most pronounced when NQO1 was present and Gem and 5’-DFUR were combined. These results suggest that tumors harboring high levels of NQO1, either as a result of the C609T variant, inducible mechanisms, or both, may have reduced proliferative and metastatic potential compared to low NQO1 expressors and be more susceptible to combination GemCap than to Gem alone.

This study has several limitations: firstly, archival material for NQO1 protein and SNP measurement was available for only a subset of ESPAC-4 patients. While, to our knowledge this subset was representative, bias may have been introduced by confounding factors unknown to us. Second, the study focused on Gem and GemCap and addressed resectable patients only. Currently for resectable and borderline resectable PDAC, the PRODIGE 24 trial recommends adjuvant mFOLFIRINOX for 24 weeks followed by surgery and chemotherapy.^29^^,^^30^ Nevertheless, GemCap remains a valuable and evidence-based choice, especially for patients with lower performance status who are not eligible for mFOLFIRINOX.^31^ Third, this research was exploratory. While the findings are promising and suggest a predictive role for NQO1 in response to GemCap therapy, they require validation and independent high-quality studies.

## Supplementary Material

djaf345_Supplementary_Data

## Data Availability

Data supporting the findings of this study are available within the paper and its supplementary information files. ESPAC data cannot be made publicly available as it contains patient information. Requests for data sharing can be made to corresponding author Eithne Costello and will be shared pending approval of the Liverpool Clinical Trial Centre Post-Trial Tissue Bank review board and appropriate human subjects protections.
