# Supplementary material for: NQO1 as a predictor of response to adjuvant GemCap treatment for pancreatic cancer
Source: J Natl Cancer Inst. 2025 Dec 1;118(4):643–54. doi: 10.1093/jnci/djaf345 (PMC13064515; doi:10.1093/jnci/djaf345)
Supplement: djaf345_Supplementary_Data [file djaf345_supplementary_data.docx]

**Supplementary Materials**

**Supplementary Methods**

**Immuno-staining and scoring of tissue micro-arrays**

Sections (4µm and 5 µm respectively) from formalin-fixed paraffin-embedded (FFPE) ESPAC tumor tissue microarrays (TMAs) or paraffin-embedded MIA PaCa-2 cell pellets were subjected to antigen retrieval. They were then washed in TBS, incubated (1 h at room temperature) with primary NQO1 Monoclonal Antibody (ThermoFisher Scientific, Invitrogen MA1-16672) diluted 1:5,000 in Dako REAL Antibody Diluent and treated with Dako EnVision+ System-HRP Labelled Polymer Anti-Mouse Secondary Antibody for 1 h at RT. Slides were counterstained with haematoxylin solution. Cores (1 to 4 per patient) were scored by specialist histopathologist FC, blinded to treatment arm, patient outcomes. The intensity of NQO1 staining in tumor cells was ranked from 0 to 4 (4 being the highest). H-scores were calculated by multiplying the intensity score by the percentage of cells stained, and mean H-scores calculated for each patient.

**Germline NQO1 SNP Analysis**

DNA from blood cells (n= 250) or formalin-fixed paraffin-embedded (FFPE) tumors (n = 127) of ESPAC-4 patients was extracted using QIAamp DNA Blood or FFPE Tissue Kits respectively (Qiagen) and the NQO1 polymorphism rs1800566 analysed using NQO1 SNP Assay (4362691, Assay ID: C_2091255_30, Thermo Scientific). Primers were combined with 5 µL DNA sample (10 ng/µl), 4 µL Nuclease-free water, 10 µL Roche LightCycler 480 Probe Master Mix (Roche LifeScience) and 1 µL ThermoFisher NQO1 SNP Assay reagent and analysed by PCR in a MicroAmp Optical 96-well Reaction Plate (Applied Biosystems) using QuantStudio 7 Flex Real-Time PCR System (Applied Biosystems) and QuantStudio Real-Time PCR Software. The expected Hardy-Weinberg equilibrium of NQO1 polymorphism rs1800566 genotype frequencies was calculated and compared to observed genotype frequencies using *X^2^* test. Allele distribution was also compared to that expected of a European cohort (gnomAD v2.1.1 (controls [20]) with a single cohort generated from a weighted mean of Finnish and non-Finnish European data. The NQO1 C609T SNP status of PDAC cell lines was evaluated through bidirectional Sanger sequencing of genomic DNA.

**In vivo experiments**

Animal experiments were performed under a UK Home Office-approved project licence. Mice were housed in a licenced establishment with free access to food and water, and under a 12-hour light-dark cycle. Drug treatments were performed in accordance with LASA best practice guidelines. Model C57BL/6J B6(Cg)-*Tyr*^c-2J^/J (also known as OKD48 [20]) mice were treated with luciferin (ProMega) via intra-peritoneal injection to generate luminescence and imaged via IVIS (PerkinElmer). Luciferin, gemcitabine, 5-FU and DPBS were delivered at 10 mL/kg in DPBS. CDDO-Me was dissolved and delivered in DMSO (2 mL/kg). Luminescence was quantified prior to drug treatment, and at 4 h, 24 h, and 1-week post-treatment. Average intensity of luminescence across the whole body and the greatest signal intensity detected from each animal were recorded. Post-treatment readings were normalised to pre-treatment readings.

**Cell Culture, drug treatments and** **In vitro assays**

**Pancreatic Cell Lines/Cell Culture**

The identity of PDAC cell lines MIA PaCa-2, PANC-1 and SUIT-2 were authenticated using short tandem repeat profiling against international reference standards and tested for mycoplasma using e-Myco plus mycoplasma PCR detection kit (iNTRON Biotechnology) following manufacturer instructions. Cells were passaged in 10% FBS (Invitrogen) Dulbecco’s Modified Eagle’s Medium (Sigma), in a humidified incubator at 37°C with 5% CO_2_. Cell counting was performed using a BioRad TC10 cell counter.

**Knockdown of Nrf2**

Transfection of siRNA was performed using lipofection. Lipofectamine (Lipofectamine^TM^ 2000 – 11668500, Invitrogen) and siRNA (Nrf2 SMARTpool: L-003755-00-0005, Dharmacon; Non-targeting: D-001220-01-05, Dharmacon) were separately diluted in Opti-MEM^TM^ I Reduced Serum Medium (11058021, Gibco) and incubated for 5 min, then combined, incubated at RT for 20 min and added dropwise to wells to final concentrations of 20 nM/40 nM siRNA. Cells were incubated for 24 h before harvest for western blot analysis. Alternatively, for NQO1 antibody validation for immunohistochemical (IHC) analysis of ESPAC samples, cells were fixed and embedded in paraffin prior to IHC staining.

**Western Blotting**

Cells were harvested in RIPA buffer (Thermo Scientific, UK) containing a final concentration of 1% protease inhibitor (Thermo Scientific, UK) and 0.1% of proteasome inhibitor MG-132 (Sigma-Aldrich). Protein (20 µg) was incubated with Reducing Sample Buffer (RSB) at 95^o^C for 10 min. Separation was performed using precast Miniprep SDS-PAGE gels (BioRad). For Nrf2 detection, gels were 7.5% polyacrylamide, otherwise Any kD™ was used. For all Nrf2 detection, electrophoresis (60V) was performed at 4°C for 30 min, then 90V until maximal separation. Otherwise, electrophoresis was performed at 300V at room temperature (RT) until maximal separation. Proteins were then transferred to PVDF membranes (BioRad) and blocked with 5% milk 0.1% PBST (Phosphate Buffered Saline; 0.1% Tris-20v/v) w/v for ≥2 h prior to incubation with primary antibody for ≥2 h. Primary antibodies and the dilutions of primary and secondary antibodies used in Western analysis were as follows: Nrf2 (ab62352, Abcam), 1° 1:1000, 2° 1:1000; NQO1 (MA1-16672, Invitrogen), 1° 1:2000, 2° 1:2000 (SUIT-2 cells), 1° 1:4000, 2° 1:4000 (MIA PaCa-2 cells); AKR1C1/2 (Abcam, ab96087) 1° 1:2000, 2° 1:2000; GCLC (Abcam, ab190685), 1° 1:1000, 2° 1:1000; β-Actin (Sigma-Aldrich, A2228), 1° 1:20,000, 2° 1:4000. Following multiple PBST washes, membranes were incubated with secondary antibody (Anti-rabbit - P0448, Dako; Anti-mouse - P0447, Dako) for 2 h and proteins revealed using Clarity Western ECL Substrate (BioRad). Chemiluminescence was measured using a BioRad Chemidoc Touch. Molecular weight analysis was performed using BioRad ImageLab software.

**Nrf2 Luciferase Assay**

A luciferase-based assay was utilised to measure Nrf2 activity. An Nrf2-inducible PGL4.11 vector containing 8 Nrf2-responsive ARE sequences was transiently transfected into MIA PaCa-2, PANC-1, SUIT-2 or HEK-293 cell lines. A solution comprising 200ng Nrf2-Luciferase plasmid, 20 ng Renilla control plasmid (E6921, ProMega) and 25 µL Opti-MEM was added per well of a white based 96-well plate (655083, Greiner Bio-One). A second solution containing 0.8µL Lipofectamine 2000 and 25 µL Opti-MEM per well was prepared and incubated at RT for 5 min before combining with the plasmid solution. The two solutions were combined and incubated for a further 30 min before adding dropwise to cells in a 96-well plate. Luciferase activity was detected using Dual-Glo® Luciferase Assay System (E2920, ProMega) according to the manufacturer’s instructions. Luminescence was measured using an integration time of 1 sec. Firefly luciferase activity was normalised to activity of the ubiquitously expressed Renilla luciferase.

**Colony Formation**

SUIT-2 and MIA PaCa-2 cells were seeded at a density of 100,000 cells/well in 6-well plates, and incubated at 37°C, 5% CO2 for 24 h. Cells were transfected with 20 nM NQO1-targetting siRNA (Dharmacon, # M-005133-02-0005) or non-targeting control siRNA (Dharmacon, # D-001206-13-05) using lipofectamine 2000 (ThermoFisher Scientific, #11668019) and Opti-MEM (Gibco, #11058021) and incubated at 37°C, 5% CO2. After 24 h, medium was removed from wells, cells were washed twice with Phosphate Buffered Saline (PBS) (ThermoFisher Scientific, #10010056) and dissociated via trypsinisation. Cells were re-seeded at 300 cells/well (for clonogenics) and 100,000 cells/well (for western blot cell lysates) in 6-well plates. After 4 h incubation, the medium in the clonogenics plates was replaced with drug-containing medium (5’-DFUR and/or gemcitabine) and incubated for up to 10 days. To confirm the NQO1 knockdown, lysates were harvested following a further 48 h incubation period, using RIPA buffer (Sigma, #R0278) containing 0.1% MG-132 proteasome inhibitor (Sigma, #474787) and 1% protease inhibitor cocktail (Sigma, #P8340). Colonies were allowed to form for 10 days before staining with glutaraldehyde/crystal violet (6.0%/0.5%) solution. After washing, plates were airdried for >12 h and then imaged.

**Statistical Methodology**

Continuous ESPAC data were summarised as median (IQR) and categorical data as frequencies of counts with associated percentages. The outcome of interest was overall survival measured as the time from randomisation until death by any cause. For overall summaries and the comparison of categorical covariates, estimates of overall survival were obtained using the Kaplan and Meier method and for continuous data the impact of overall survival was evaluated using weighted Kernel Estimators. The impact of biomarkers and other prognostic covariates on overall survival was evaluated using Cox Proportional hazards models. Multivariable models were constructed using a backwards stepwise procedure based on Akaikes Information Criterion (AIC). Upon the construction of main effects models, the impact of biomarkers was included as nested effects within treatment groups to investigate if biomarkers can be considered as predictive as opposed to prognostic. NQO1 was modelled as a continuous covariate to avoid loss of information. Where necessary, NQO1 was dichotomised to visualise the impact of the covariates on outcomes. Results are presented in terms of Hazard Ratios with 95% confidence intervals. All analyses were conducted using R (Version 4.1). For clonogenic analysis, colony intensity percentages were determined through ImageJ (Version 1.54f), using the ColonyArea plugin [20]. For all other analysis, statistical tests were performed using R (Version 4.3.0) and GraphPad Prism (Version 6.01).

**Supplementary figures**


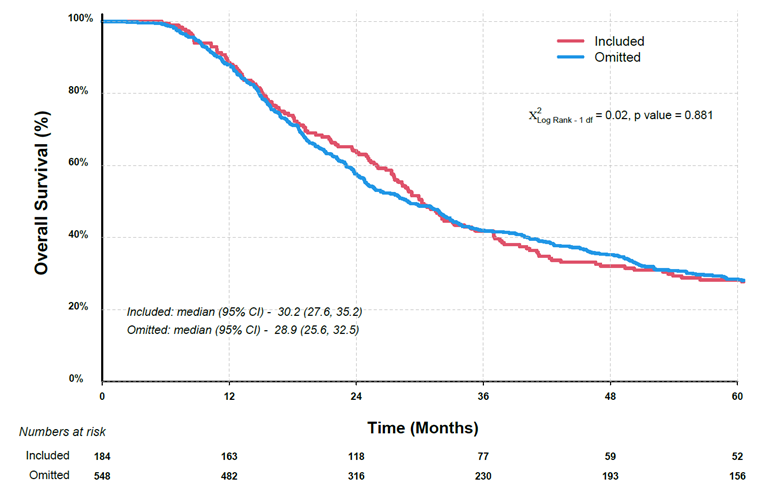


**Figure S1: Survival analyses.** Kaplan Meier plot showing overall survival of ESPAC-4 patients included (i.e. NQO1 levels determined) or omitted in this study. No significant survival difference was observed.


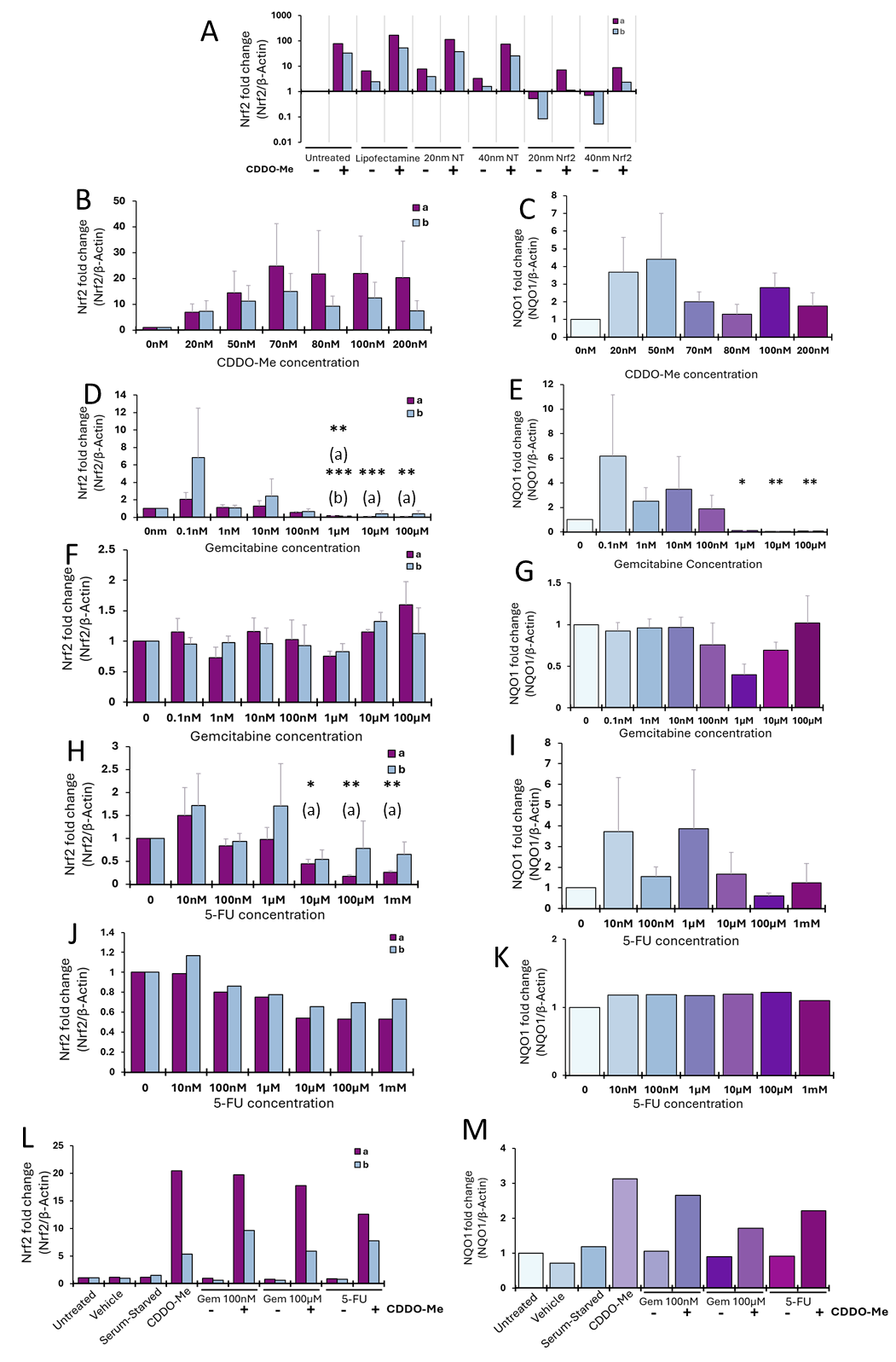


**Figure S2. Densitometric analysis of Nrf2 (bands a and b) and NQO1 protein bands corresponding to the representative immunoblots shown in Figure 4A-G.** Band intensities were quantified and normalised to the untreated/vehicle control for each experiment. **(A)** Quantification of Nrf2 (n=1) from Figure 4A. **(B, C)** Quantification of Nrf2 and NQO1 (n=3), respectively, of repeats of Figure 4B. **(D, E)** Quantification of Nrf2 and NQO1 (n=3), respectively, of repeats of Figure 4C. **(F, G)** Quantification of Nrf2 and NQO1 (n=3), respectively, of repeats of Figure 4D. **(H, I)** Quantification of Nrf2 and NQO1 (n=3), respectively, of repeats of Figure 4E. **(J, K)** Quantification of Nrf2 and NQO1, respectively, from Figure 4F. **(L, M)** Quantification of Nrf2 and NQO1, respectively, from Figure 4G. Significance was determined via student’s t-test. Error bars + SEM. * = P < 0.05, ** = P < 0.01, *** = P < 0.001. Parentheses indicate which Nrf2 band asterisks applies to.

**
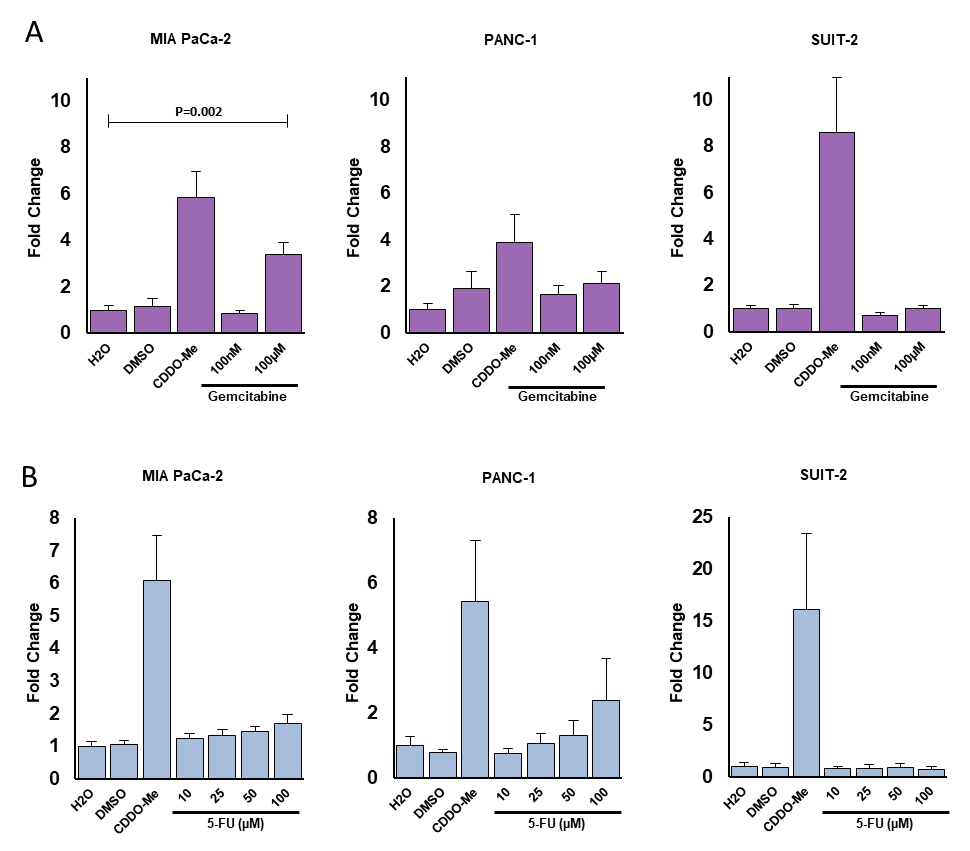
**

**Figure S3:** **Nrf2 activity in response to Gemcitabine and 5-FU**

Luminescence readings of MIA PaCa-2, PANC-1 and SUIT-2 cells treated for 24 hours with **A** gemcitabine (Gem) and **B** 5-FU following transient transfection of an Nrf2-luciferase reporter plasmid. CDDO-Me was used as a positive control. DMSO and water were used as vehicle controls for gemcitabine/5-FU and CDDO-Me, respectively. Data represent the mean of three independent experiments, each performed in triplicate. Significance was determined via one-way ANOVA with Bonferroni’s multiple comparison test and independent t-test (P = 0.002). Error bars + SEM.

**Figure S4:** **NQO1 protein levels following 24 h treatment with gemcitabine and 5’-DFUR.**

Representative Western blot images showing expression of indicated proteins in PANC-1 (**A**), and MIA PaCa-2 (**B**) cell lysates 24 hours post-treatment with increasing concentrations of 5’-DFUR. The Nrf2 inducer CDDO-Me was included as a positive control, while vehicle-only treatment served as a negative control (-ve) (all n = 3). β-Actin was used as a loading control. **C-D** Quantification of NQO1 band intensity for PANC-1 (**C**), and MIA PaCa-2 (**D**), normalized to β-actin and expressed as a percentage of the negative control. **E–F** Western blot images of MIA PaCa-2 (n = 2) (**E**) cell lysates treated with increasing concentrations of 5’-DFUR, in combination with a fixed concentration of gemcitabine (1 µM) with NQO1 quantification (**F**), normalized to β-actin and expressed as a percentage of the negative control. Significant differences relative to the -VE control were determined via Mann-Whitney U tests with Benjamini-Hochberg multiple comparison corrections. Error bars + SEM.

**Figure S5: Effects of NQO1 knockdown and drug treatments on colony formation**

**A.** Evaluation of the effect of NQO1 knockdown on colony formation in MIA PaCa-2 cells; (n=2) after 24 h incubation with non-targeting or NQO1-targeting siRNA, re-seeding and incubation up to 10 days. **B.** Average Colony Intensity Percentages presented for MIA PaCa-2 cells (from **A**). **C.** Evaluation of the effect of indicated drugs combined with NQO1 knockdown on colony formation in MIA PaCa-2 cells (seeded at 300 cells/well) **D.** Column chart of Average Colony Intensity Percentage of MIA PaCa-2 cells (from **C**). H_2_O and DMSO are respective vehicle controls for gemcitabine and 5’-DFUR. G+D = combined gemcitabine and 5’-DFUR. Two-way ANOVA with Tukey’s post hoc test was used to assess inter-group and inter-treatment differences (vehicle controls excluded). Error bars + SEM. * = P < 0.05, ** = P < 0.01, ***= P < 0.001.

**Figure S6: Effects of NQO1 knockdown and drug treatments on colony formation.**

**A** Western blot analysis of SUIT-2 cell lysates 48h post-treatment with non-targeting or NQO1-targeting siRNA. **B.** Evaluation of the effect of indicated drugs combined with NQO1 knockdown. After 24 h incubation with non-targeting or NQO1-targeting siRNA, cells were re-seeded (500 cells/well) and 4 h later the medium replaced with drug-containing medium (5’-DFUR and/or gemcitabine) and cells incubated for up to 10 days. **C.** Column chart of Average Colony Intensity Percentage of Suit-2 cells (from **B**). H_2_O and DMSO are respective vehicle controls for gemcitabine and 5’-DFUR. **D-E.** Mean number of colonies (**D**) and mean colony diameter (µM) (**E**) of MIA PaCa-2 cells (from Figure 6E). **F-G.** Mean number of colonies (**F**) and mean colony diameter (µM) (**G**) of SUIT-2 cells (from C above). G+D = combined gemcitabine and 5’-DFUR. Two-way ANOVA with Tukey’s post hoc test was used to assess inter-group and inter-treatment differences (vehicle controls excluded). Error bars + SEM. * = P < 0.05, ** = P < 0.01, ***= P < 0.001.

**Supplementary Tables**

**Table S1. Comparison of ESPAC patients included in this study (where NQO1 tumor protein expression was determined) versus those omitted.**

| Covariate | Level | Included (%) | Omitted (%) | Total |
| --- | --- | --- | --- | --- |
| Total number |  | 184 | 548 | 732 |
| Sex | Female | 96 (52%) | 319 (58%) | 415 (57%) |
| Sex | Male | 88 (48%) | 229 (42%) | 317 (43%) |
| Sex | Missing | 0 (0%) | 0 (0%) | 0 (0%) |
| Maximum Tumor Size | Median (IQR) | 30 (25, 37) | 30 (24, 40) | 30 (24, 40) |
| Maximum Tumor Size | Missing | 1 | 15 | 16 |
| Resection margin | Negative | 118 (64%) | 321 (59%) | 439 (60%) |
| Resection margin | Positive | 66 (36%) | 227 (41%) | 293 (40%) |
| Resection margin | Missing | 0 (0%) | 0 (0%) | 0 (0%) |
| Nodal Status | Negative | 153 (83%) | 435 (79%) | 588 (80%) |
| Nodal Status | Positive | 31 (17%) | 113 (21%) | 144 (20%) |
| Nodal Status | Missing | 0 (0%) | 0 (0%) | 0 (0%) |
| WHO Performance Status | 0 | 82 (45%) | 226 (41%) | 308 (42%) |
| WHO Performance Status | 1 | 99 (54%) | 304 (55%) | 403 (55%) |
| WHO Performance Status | 2 | 3 (2%) | 18 (3%) | 21 (3%) |
| WHO Performance Status | Missing | 0 (0%) | 0 (0%) | 0 (0%) |
| Tumour Differentiation | Well | 18 (10%) | 76 (14%) | 94 (13%) |
| Tumour Differentiation | Moderate | 118 (64%) | 328 (60%) | 446 (61%) |
| Tumour Differentiation | Poor | 48 (26%) | 141 (26%) | 189 (26%) |
| Tumour Differentiation | Missing | 0 (0%) | 3 (1%) | 3 (0%) |
| Smoking Status | No | 67 (36%) | 181 (33%) | 248 (34%) |
| Smoking Status | Past | 57 (31%) | 189 (34%) | 246 (34%) |
| Smoking Status | Present | 24 (13%) | 79 (14%) | 103 (14%) |
| Smoking Status | Missing | 36 (20%) | 99 (18%) | 135 (18%) |
| Diabetic Status | Insulin Dependent | 23 (12%) | 60 (11%) | 83 (11%) |
| Diabetic Status | No | 114 (62%) | 334 (61%) | 448 (61%) |
| Diabetic Status | Not Insulin Dependent | 17 (9%) | 65 (12%) | 82 (11%) |
| Diabetic Status | Missing | 30 (16%) | 89 (16%) | 119 (16%) |
| Treatment | GEM | 94 (51%) | 273 (50%) | 367 (50%) |
| Treatment | GEMCAP | 90 (49%) | 275 (50%) | 365 (50%) |
| Treatment | Missing | 0 (0%) | 0 (0%) | 0 (0%) |
| Post-Operative CA199 | median (IQR) | 17.3 (10, 55.75) | 19.2 (9, 55.125) | 19 (9, 55.175) |
| Post-Operative CA199 | Missing | 18 | 48 | 66 |
| NQO1 H-score | Missing | 0 | 548 | 548 |

 For categorical variables, the number of patients is provided, alongside proportions of the overall sample in parentheses. WHO: World Health Organization (WHO)

**Table S2 Comparison of populations with CC and CT genotypes. Note TT, though rare, are included for completeness.**

| Covariate | Level | CC | CT | TT | Missing | Total |
| --- | --- | --- | --- | --- | --- | --- |
| Total number |  | 125 | 54 | 2 | 3 | 184 |
| Sex | F | 66 (53%) | 29 (54%) | 0 (0%) | 1 (33%) | 96 (52%) |
| Sex | M | 59 (47%) | 25 (46%) | 2 (100%) | 2 (67%) | 88 (48%) |
| Sex | Missing | 0 (0%) | 0 (0%) | 0 (0%) | 0 (0%) | 0 (0%) |
| Max. Tumor Size | median (IQR) | 30 (22, 35) | 30 (27, 37) | 45 (42.5, 47.5) | NA | 30 (25, 37) |
| Max. Tumor Size | Missing | 0 | 1 | 0 | 0 | 1 |
| Resection margin | Negative | 78 (62%) | 37 (69%) | 2 (100%) | 1 (33%) | 118 (64%) |
| Resection margin | Positive | 47 (38%) | 17 (31%) | 0 (0%) | 2 (67%) | 66 (36%) |
| Resection margin | Missing | 0 (0%) | 0 (0%) | 0 (0%) | 0 (0%) | 0 (0%) |
| Nodal Status | Negative | 104 (83%) | 45 (83%) | 2 (100%) | 2 (67%) | 153 (83%) |
| Nodal Status | Positive | 21 (17%) | 9 (17%) | 0 (0%) | 1 (33%) | 31 (17%) |
| Nodal Status | Missing | 0 (0%) | 0 (0%) | 0 (0%) | 0 (0%) | 0 (0%) |
| WHO | 0 | 58 (46%) | 22 (41%) | 2 (100%) | 0 (0%) | 82 (45%) |
| WHO | 1 | 65 (52%) | 31 (57%) | 0 (0%) | 3 (100%) | 99 (54%) |
| WHO | 2 | 2 (2%) | 1 (2%) | 0 (0%) | 0 (0%) | 3 (2%) |
| WHO | Missing | 0 (0%) | 0 (0%) | 0 (0%) | 0 (0%) | 0 (0%) |
| Differentiation | Well | 11 (9%) | 6 (11%) | 0 (0%) | 1 (33%) | 18 (10%) |
| Differentiation | Moderate | 80 (64%) | 36 (67%) | 1 (50%) | 1 (33%) | 118 (64%) |
| Differentiation | Poor | 34 (27%) | 12 (22%) | 1 (50%) | 1 (33%) | 48 (26%) |
| Differentiation | Missing | 0 (0%) | 0 (0%) | 0 (0%) | 0 (0%) | 0 (0%) |
| Smoking Status | No | 42 (34%) | 23 (43%) | 1 (50%) | 1 (33%) | 67 (36%) |
| Smoking Status | Past | 38 (30%) | 17 (31%) | 0 (0%) | 2 (67%) | 57 (31%) |
| Smoking Status | Present | 16 (13%) | 8 (15%) | 0 (0%) | 0 (0%) | 24 (13%) |
| Smoking Status | Missing | 29 (23%) | 6 (11%) | 1 (50%) | 0 (0%) | 36 (20%) |
| Diabetic Status | Insulin Dependent | 15 (12%) | 7 (13%) | 0 (0%) | 1 (33%) | 23 (12%) |
| Diabetic Status | No | 76 (61%) | 36 (67%) | 0 (0%) | 2 (67%) | 114 (62%) |
| Diabetic Status | Not Insulin Dependent | 11 (9%) | 5 (9%) | 1 (50%) | 0 (0%) | 17 (9%) |
| Diabetic Status | Negative | 23 (18%) | 6 (11%) | 1 (50%) | 0 (0%) | 30 (16%) |
| Treatment | GEM | 66 (53%) | 25 (46%) | 2 (100%) | 1 (33%) | 94 (51%) |
| Treatment | GEMCAP | 59 (47%) | 29 (54%) | 0 (0%) | 2 (67%) | 90 (49%) |
| Treatment | Missing | 0 (0%) | 0 (0%) | 0 (0%) | 0 (0%) | 0 (0%) |
| Post-Operative CA199 | median (IQR) | 17 (9.375, 55.25) | 21 (10, 61) | 47 (27.5, 66.5) | NA | 17.3 (10, 55.75) |
| Post-Operative CA199 | Missing | 13 | 5 | 0 | 0 | 18 |
| NQO1 H-score | median (IQR) | 160 (103.75, 217.5) | 100.833 (51.562, 177.917) | 2.5 (1.25, 3.75) | NA | 139.375 (92.188, 202.812) |

 For categorical variables, the number of patients is provided, alongside proportions of the overall sample in parentheses. WHO: World Health Organization (WHO).

**Table S3. Adverse event summary table.** Summarising the number of patients experiencing adverse events and the results of univariable logistic regressions.

| Variable | Category | Adverse Events Absent (N = 308) | Adverse Events  Present (N = 399) | Overall  (N = 707) | Univariable Logistic Regression | |
| --- | --- | --- | --- | --- | --- | --- |
|  |  |  |  |  | Odds Ratio (95% CI) | P-value |
| Sex |  |  |  |  |  |  |
|  | Female | 175 (56.8%) | 228 (57.1%) | 403 (57.0%) | - | - |
|  | Male | 133 (43.2%) | 171 (42.9%) | 304 (43.0%) | 0.987 (0.731,1.333) | 0.931 |
| Smoking Status |  |  |  |  |  |  |
|  | No | 107 (34.7%) | 135 (33.8%) | 242 (34.2%) | - | - |
|  | Past | 104 (33.8%) | 134 (33.6%) | 238 (33.7%) | 1.021 (0.712,1.465) | 0.909 |
|  | Present | 36 (11.7%) | 62 (15.5%) | 98 (13.9%) | 1.365 (0.846,2.226) | 0.206 |
|  | Missing | 61 (19.8%) | 68 (17.0%) | 129 (18.2%) |  |  |
| Diabetic Status |  |  |  |  |  |  |
|  | No | 193 (62.7%) | 241 (60.4%) | 434 (61.4%) | - | - |
|  | Not Insulin Dependent | 25 (8.1%) | 52 (13.0%) | 77 (10.9%) | 1.666 (1.007,2.821) | 0.051 |
|  | Insulin Dependent | 34 (11.0%) | 47 (11.8%) | 81 (11.5%) | 1.107 (0.687,1.8) | 0.678 |
|  | Missing | 56 (18.2%) | 59 (14.8%) | 115 (16.3%) | - | - |
| WHO Performance Status |  |  |  |  |  |  |
|  | 0 | 140 (45.5%) | 157 (39.3%) | 297 (42.0%) | - | - |
|  | 1 | 163 (52.9%) | 227 (56.9%) | 390 (55.2%) | 1.242 (0.916,1.684) | 0.163 |
|  | 2 | 5 (1.6%) | 15 (3.8%) | 20 (2.8%) | 2.675 (1.008,8.396) | 0.063 |
| Tumour Differentiation |  |  |  |  |  |  |
|  | 1 | 42 (13.6%) | 51 (12.8%) | 93 (13.2%) | - | - |
|  | 2 | 178 (57.8%) | 251 (62.9%) | 429 (60.7%) | 1.161 (0.737,1.822) | 0.516 |
|  | 3 | 86 (27.9%) | 96 (24.1%) | 182 (25.7%) | 0.919 (0.555,1.517) | 0.742 |
|  | Missing | 2 (0.6%) | 1 (0.3%) | 3 (0.4%) | - | - |
| Maximum Tumour Size |  |  |  |  |  |  |
|  | Mean (SD) | 32.2 (12.6) | 32.6 (14.5) | 32.4 (13.7) | 1.031 (0.887,1.201) | 0.691 |
|  | Median (Q1,Q3) | 30.0 (25.0,37.0) | 30.0 (24.0,40.0) | 30.0 (24.0,40.0) | - | - |
|  | Missing | 7 (2.3%) | 8 (2.0%) | 15 (2.1%) | - | - |
| Post-Operative CA 19-9 |  |  |  |  |  |  |
|  | Mean (SD) | 152 (655) | 108 (365) | 127 (512) | 0.997 (0.915,1.087) | 0.95 |
|  | Median (Q1,Q3) | 18.0 (6.00,47.3) | 16.1 (7.10,46.0) | 17.0 (7.00,46.5) | - | - |
| Lymph Node Positivity |  |  |  |  |  |  |
|  | Negative | 66 (21.4%) | 76 (19.0%) | 142 (20.1%) | - | - |
|  | Positive | 242 (78.6%) | 323 (81.0%) | 565 (79.9%) | 1.159 (0.8,1.677) | 0.434 |
| NQO1 Genotype |  |  |  |  |  |  |
|  | CC | 74 (24.0%) | 112 (28.1%) | 186 (26.3%) | - | - |
|  | CT | 38 (12.3%) | 43 (10.8%) | 81 (11.5%) | 0.748 (0.442,1.267) | 0.278 |
|  | Missing | 196 (63.6%) | 244 (61.2%) | 440 (62.2%) | - | - |
| NQO1 H-Score |  |  |  |  |  |  |
|  | >150 | 40 (13.0%) | 44 (11.0%) | 84 (11.9%) | - | - |
|  | <150 | 38 (12.3%) | 62 (15.5%) | 100 (14.1%) | 1.48 (0.822, 2.665) | 0.244 |
|  | Missing | 230 (74.7%) | 293 (73.4%) | 523 (74.0%) | - | - |
| Treatment |  |  |  |  |  |  |
|  | Gemcitabine | 172 (55.8%) | 184 (46.1%) | 356 (50.4%) | - | - |
|  | GEMCAP | 136 (44.2%) | 215 (53.9%) | 351 (49.6%) | 1.478 (1.097,1.994) | 0.01** |

For categorical variables, the number of patients experiencing adverse events in each category is summarised, alongside proportions of the overall sample in parentheses. For continuous variables, patients experiencing adverse events are summarised by means with standard deviations in parentheses, and medians with interquartile ranges in parentheses. Results of univariable logistic regressions for each variable are presented as odds ratios with 95% confidence intervals in parentheses, alongside p-values. Continuous variables were z-transformed for modelling purposes, other than CA 19-9, which was log(+1) transformed and had a dummy variable denoting missing CA 19-9 data nested within it. Significant P-values are denoted by **.
